# Supplementary material for: Ultrasound-Mediated Drug Diffusion, Uptake, and Cytotoxicity in a Glioblastoma 3D Tumour Sphere Model
Source: Cells. 2025 Jun 11;14(12):886. doi: 10.3390/cells14120886 (PMC12191014; doi:10.3390/cells14120886)
Supplement: Supplementary file 1 [file cells-14-00886-s001.zip › cells-3616389-supplementary.pdf]

Supplementary information

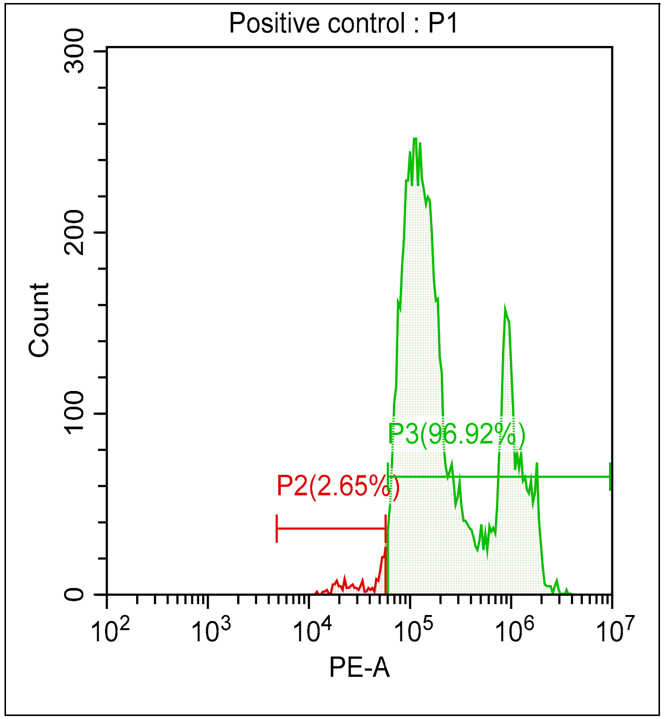

Figure S1: Positive control: PI uptake in 20% DMSO-treated U-251 MG tumour spheres.

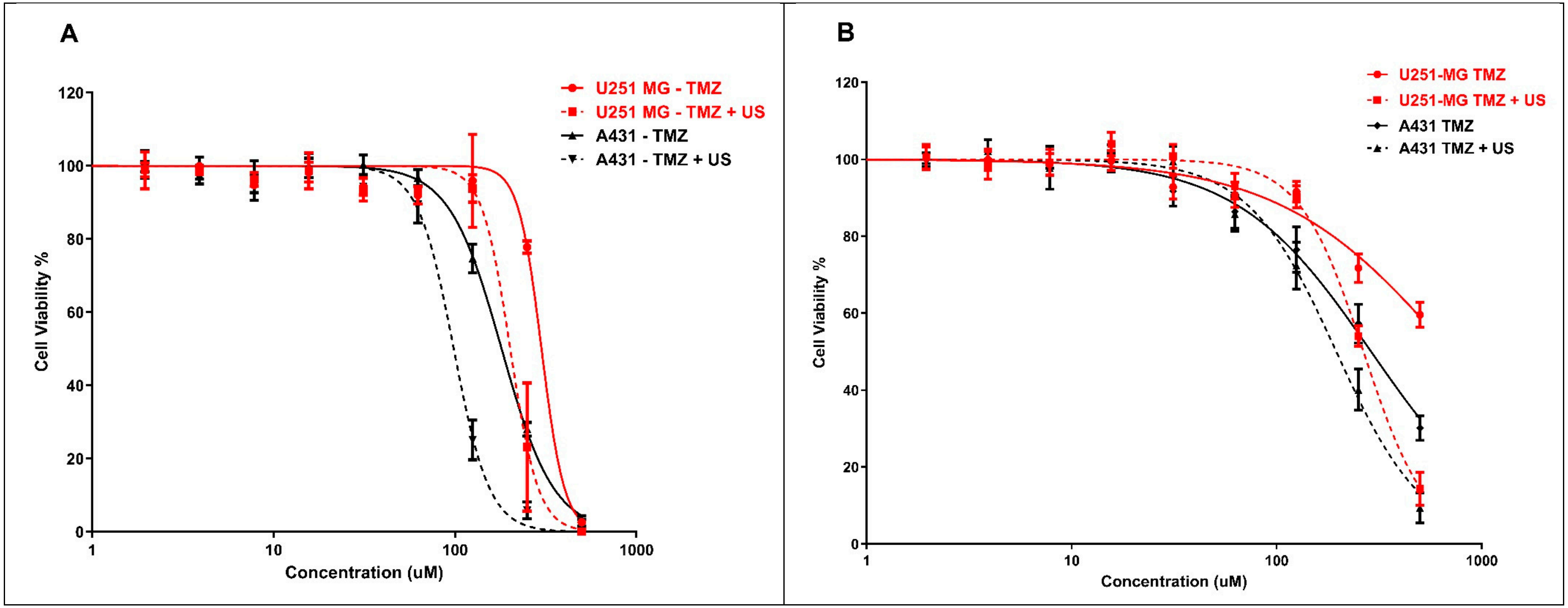

Figure S2: TMZ cytotoxicity analysis with and without US (3 min) combination using U-251 MG and A431 2D cells and 3D tumour spheroids A) TMZ cytotoxicity in 2D cell cultures after 48h incubation. B) TMZ cytotoxicity in 3D cell cultures after 48h incubation.

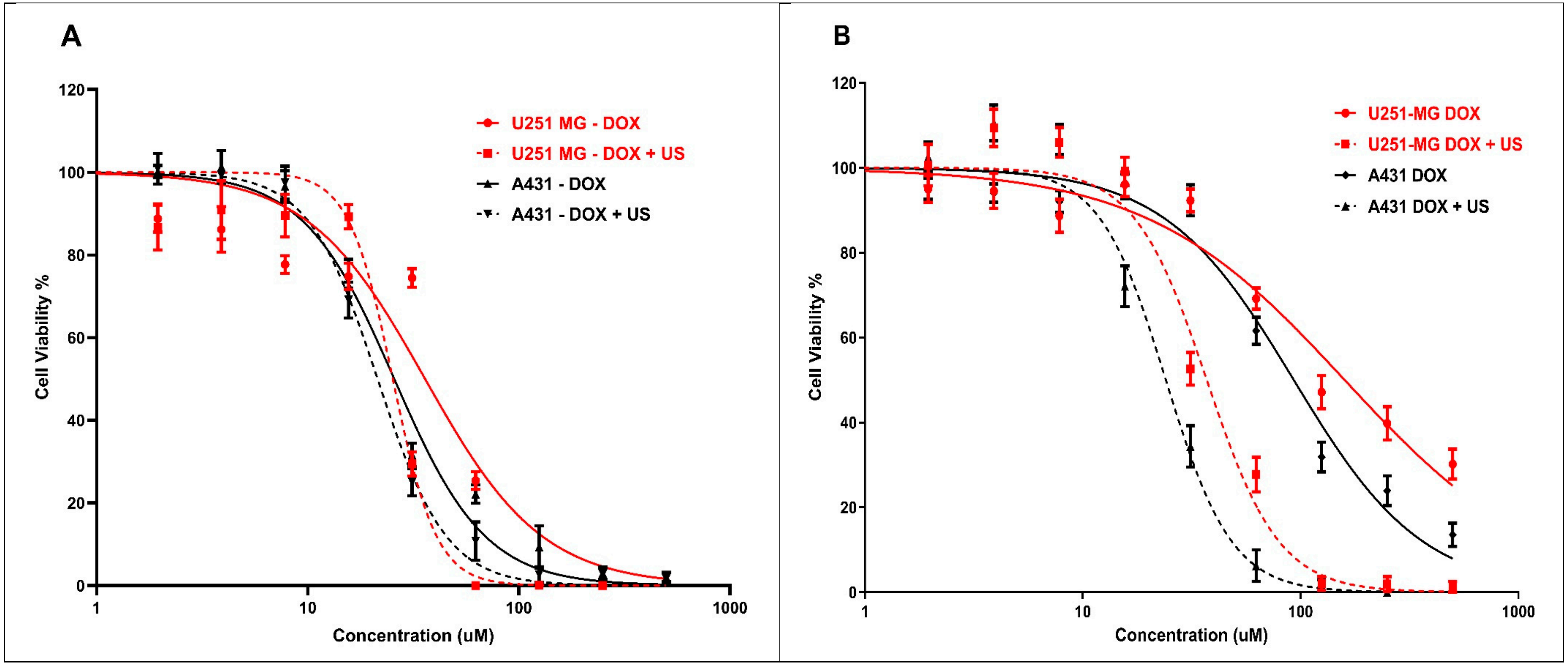

Figure S3: DOX cytotoxicity analysis with and without US (3 min) combination using U-251 MG and A431 2D cells and 3D tumour spheres. A) DOX cytotoxicity in 2D cells with 48h incubation. B) DOX cytotoxicity in U-251 MG and A431 3D cells with 48h incubation.

TableS1. IC50 Values and ranges of, U-251 MG single and multiple US treatment with different incubations.

**US post treatment incubation**	**Treatment frequency**	**IC_{50} (min)**	**IC_{50} Range**	**Hillslope**	**Figure**

8h	1X	75.22	58.16 to 97.29	0.1079	3A
	2X	47.25	40.76 to 54.78	0.1173	3A
	3X	33.55	30.93 to 36.39	0.05671	3A
	4X	27.91	26.98 to 28.86	0.03511	3A
	5X	26.41	25.55 to 27.31	0.07038	3A
24h	1X	24.87	24.35 to 25.39	0.02533	3B
	2X	20.35	19.66 to 21.06	0.03761	3B
	3X	11.91	11.77 to 12.05	0.01649	3B
	4X	10.39	10.23 to 10.56	0.02537	3B
	5X	9.186	9.080 to 9.294	0.01730	3B
48h	1X	30.95	29.55 to 32.42	0.02445	3C
	2X	28.93	27.28 to 30.68	0.02901	3C
	3X	16.36	15.98 to 16.74	0.01490	3C
	4X	12.11	11.88 to 12.34	0.01912	3C
	5X	7.907	7.776 to 8.041	0.02260	3C
72h	1X	57.91	44.60 to 75.20	0.09236	3D
	2X	39.33	36.27 to 42.65	0.02985	3D
	3X	26.21	24.95 to 27.53	0.02611	3D
	4X	17.15	16.70 to 17.62	0.02527	3D
	5X	8.468	8.333 to 8.605	0.02142	3D

96h	1X	42.73	38.81 to 47.05	0.05389	3E
	2X	39.30	35.70 to 43.27	0.04173	3E
	3X	17.54	17.03 to 18.07	0.01626	3E
	4X	12.52	12.27 to 12.77	0.02337	3E
	5X	7.969	7.853 to 8.086	0.02794	3E
120h	1X	15.48	15.10 to 15.87	0.02266	3F
	2X	16.90	16.46 to 17.34	0.03330	3F
	3X	10.12	9.873 to 10.37	0.03132	3F
	4X	9.154	8.969 to 9.342	0.02958	3F
	5X	6.002	5.883 to 6.124	0.03623	3F
